# Regional Pericarditis or an Alternate Diagnosis?

**DOI:** 10.1155/2014/313607

**Published:** 2014-06-26

**Authors:** Lovely Chhabra, Mohammadtokir Mujtaba, David H. Spodick

**Affiliations:** ^1^Department of Cardiovascular Medicine, Hartford Hospital, University of Connecticut School of Medicine, 80 Seymour Street, Hartford, CT 06102, USA; ^2^Department of Cardiovascular Medicine, Saint Vincent Hospital, University of Massachusetts Medical School, Worcester, MA 01608, USA

We read the paper by Alhammouri and Omar published in a recent issue of the Case Reports in Medicine [1]. The authors have described a case of what appeared to be a case of localized pericarditis; however, we wonder if alternate diagnosis was considered in the differential diagnosis. The initial ECG illustration shows ST-elevation localized to the inferior leads with reciprocal ST-depressions in V1–V3 which is usually quite unusual for acute pericarditis [2]. Spodick's sign has traditionally been defined in prior investigations by the senior author as an electrocardiographic clue to help differentiating between acute pericarditis and acute myocardial infarction; however, to best of our knowledge, this is usually valid in absence of reciprocal ST-depressions [2–6]. The pathophysiogenesis of the Spodick's sign is likely related to the diastolic injury current. During diastole, the injured subepicardial zone remains partly polarized (probably due to reduced resting transmembrane potential), as in systole. Thus, there is a potential difference between it and the completely polarized remaining myocardial mass, producing a diastolic injury current oppositely oriented to the systolic injury current that shifts electrical diastole, the TQ interval. This interval corresponds to the ECG baseline (TP) and cannot be detected by the clinical ECG so that it appears as a J (ST) shift in the opposite direction [2].

Similarly, authors have used the term “reverse Spodick's sign” which merely simulates reciprocal ST-depressions; however, in our opinion, it needs a validation in large controlled investigations if indeed it is associated with acute localized pericarditis. Cardiac magnetic resonance imaging would have been helpful in this case for diagnostic confirmation [7].

Also, the authors have mentioned normal right coronary and left anterior descending arteries. What about left circumflex artery: was that also normal? Notably, the second ECG obtained after a few hours shows complete normalization of prior ST-T changes without any use of anti-inflammatory therapies (NSAID's or Colchicine) and, in fact, did not demonstrate dynamic ECG changes of four-stage pericarditis [2, 3, 7]. This makes us wonder if other potential explanation such as transient coronary vasospasm may serve as an explanation for the noted clinical and ECG changes on the initial presentation.
